# Effects of nitric oxide in mucociliary transport

**DOI:** 10.1016/S1808-8694(15)30551-6

**Published:** 2015-10-19

**Authors:** Eleonora Elisia Abra Blanco, Marli Cardoso Martins Pinge, Otavio André Andrade Neto, Nathália Gardin Pessoa

**Affiliations:** 1 Doctorate degree. Associate faculty member; 2 Post-doctoral degree. Associate faculty member; 3 Biomedical specialist. Undergraduate course in biomedicine; 4 Pharmacy and biochemistry course student. Londrina Federal University

**Keywords:** mucociliary clearance, enzyme inhibitors, respiratory mucosa

## Abstract

The airways are made up of ciliated epithelium which secretes mucous, protecting the respiratory tract from particles inhaled during breathing. Its is paramount to understand the physiology and the mechanisms involved in mucociliary activity. Literature suggests that Nitric oxide (NO), especially the one produced by iNOS expression, maintains the mucociliary function and the immune defense of the nasal cavity.

**Aim:**

to assess NO participation and the enzymatic pathways in the production of NO and mucociliary transport, using constructive and inductive NO synthetase inhibitors, L-NAME and aminoguanidine, respectively.

**Materials and methods:**

frog palates were prepared and immerse in ringer (control), L-NAME or aminoguanidine solutions. The palates were immerse in these solutions for four periods of 15 minutes. Mucociliary transport measures were carried out before and after each exposure.

**Results:**

control palates maintained stable their transportation speed. L-NAME increased, while aminoguanidine reduced mucous transportation velocity.

**Conclusion:**

unspecific cNOS block with L-NAME and relatively specific iNOS block with aminoguanidine results leads us to propose that depending on the pathway, the NO can increase or reduce mucociliary transport in frog palates.

## INTRODUCTION

The respiratory system is the first inner region of the body to make contact with the external environment. Airways are the first interface between the internal milieu and microorganisms, allergens, or inhaled particles. A variety of particles and chemical substances are deposited in the respiratory apparatus during breathing.

The respiratory tract has a sophisticated defense mechanism, the mucociliary apparatus, to support the homeostasis of this delicate system.^1^ The airway mucosa, from the nasal cavity to the bronchioles, consists of pseudostratified and ciliated epithelium interspaced by submucosal glands and goblet cells, which produce mucus.

Mucociliary transport is an important defense mechanism for the respiratory mucosa; it removes inhaled particles from this surface. The driving force for this complex system is the ciliary activity on the respiratory epithelium, commonly quantified by the ciliary beat frequency^2^ a measure of mucociliary transport velocity.^3^

The efficiency of mucociliary transport depends mainly on: the thickness of the mucus layer and the composition and rheological properties of mucus; cilia in cells with perfectly preserved structures, to efficiently perform ciliary movement; coordination among adjacent cilia to yield a beat wave for propulsion of mucus.^4^, ^5^ Failure of the ciliary system may result in: easier bacterial colonization, thus increasing the risk of respiratory infections; increased contact time between harmful agents and the respiratory epithelium; stasis of mucus that may result in ventilation disorders and increased airflow resistance.^6^ Thus, mucociliary transport dysfunction may worsen the quality of life of individuals. It is therefore crucial to understand the physiology of the mucociliary apparatus, how its components affect the clearance of respiratory secretions, and what are the control mechanisms and the actions of endogenous substances on mucociliary activity.

Seromucus glands are located in the submucosa in the nasal cavity. Goblet cells are prevalent in the paranasal sinus.^7^ Nasal mucus consists of goblet cell, submucosal gland and lacrimal gland secretions mixed with water. The composition of mucus is altered in pathological conditions, which directly or indirectly affect mucociliary function.^8^

The autonomic system is the most important nasal secretion control mechanism; parasympatic stimuli increase secretion volume. Other non-acetylcholine-mediated mechanisms, however, have been suggested.^8^

The lungs are the main nitric oxide (NO) producing site in the blood circulatory system.^9^ NO is produced from L-arginine (L-Arg) in airways and is a component of physiological and pathophysiological events such as vasodilatation, broncodilatation, neurotransmission and bacteriostasis.^10^ NO production is set in motion by the NO synthase enzyme (NOS). There are three distinct isoforms of NOS: endothelial NOS (eNOS), neuronal NOS (nNOS), both of which are expressed constitutively and named cNOS, and an inductive pathway (iNOS). These three NOS isoforms may be found in the respiratory tract, and add to NO production.^11^ It has been shown that NO production takes place due to continuous expression of iNOS in epithelial cells within the airways of healthy individuals. This enzyme appears to have a crucial role in defending the airways against infection.^12^

Topical application of NG-nitro-L-arginine-methylester (L-NAME), a cNOS inhibitor, reduced nasal NO concentrations. Topical application of sodium nitroprussiate, an NO donor, increased nasal NO and decreased the transport time of nasal saccharine, which is an indicator of mucociliary function. Additionally, L-NAME prolonged the transport time. These observations suggested that artificially changing nasal NO production could affect mucociliary function.^13^

The concentration of nasal NO is decreased in acute and chronic rhinosinusitis, cystic fibrosis, primary ciliary dysfunction, chronic coughing, and exposure to tobacco and alcohol; these conditions are characterized by altered upper airway ciliary mucosal histology.^14^ Low NO production due to iNOS in the maxillary sinuses of rhinosinusitis and septicemia patients has been associated with a decreased function of defense mechanisms and an increased risk of secondary infections.^15^

It has been suggested that NO production due to iNOS is increased in the nasal epithelium of allergic rhinitis patients. A study of acetylcholine and L-NAME showed that although baseline nasal ciliary activity depends on endogenous NO production, cilia may be stimulated by cholinergic^16^ or mechanical^17^ stimuli regardless of endogenous NO production. For these two stimuli, the joint action of NO on ciliary beats improves local defense against allergens in allergic rhinitis patients.

Cilia move by using energy stored as ATP in mitochondria; thus, cilia continue to move even when the blood supply is interrupted, as long as local conditions are favorable, until all ATP is consumed. A convenient system for studying mucociliary transport is the frog palate; it is ciliated and secretes mucus similar to what is found in human airways.^1^, ^3^

The purpose of this study was to assess the role of NO in regulating ciliary transportability under normal conditions. The effects of NO producing constitutive and inductive enzyme inhibitors on mucociliary transportability in frog palates were studied with the aim of observing how NO participated in this mechanisms and characterizing the NO production pathway involved with mucociliary transportability.

## MATERIAL AND METHODS

The sample consisted of 85 adult frogs of both sexes of the species Rana catesbiana, weighing from 90 to 150 g, acquired from a commercial frog farm. The frogs were kept in appropriate boxes at 23°C±2.0. The palates were harvested by placing the frogs in cold water until sensitivity to mechanical stimuli was lost, after which they were sacrificed by decapitation; the mandible was then disarticulated and the upper portion (palate) was separated. Palates were kept at 4°C in a closed chamber for 72 hours to deplete the mucus. Palates were removed from the refrigerator 20 minutes before the experiment and placed in an acrylic chamber coupled to an ultrasound nebulizer to keep the environment within the chamber at 100% humidity; they were then saturated with a modified Ringer's solution for frogs (one part of Ringer's solutions and one part of distilled water - RingerR). During this period, the palate temperature increased to ambient temperature; the temperature inside the acrylic chamber was 24°C. Samples of mucus were taken from the posterior portion of the palates and immediately immersed in mineral oil to avoid dehydration. Under such experimental conditions, the mucus layer is depleted, but ciliary activity remains.^18^, ^1^

There were nine experimental groups of frog palates in this study. Five groups were used for assessing NO action (due to iNOS activation) on mucociliary transport velocity. Aminoguanidine was used for blocking iNOS in four groups comprising palates immersed in RingerR-diluted aminoguanidine solutions at 40 ppm (n=8), 50 ppm (n=10), 60 ppm (n=10) and 80 ppm (n=8); there was a control group (n=9) consisting of palates immersed in a RingerR solution only. The action of NO (due to cNOS activation) on mucociliary transport velocity was assessed in the other four groups of palates. Non-specific blockage of this pathway was attained by using L-NAME in three groups of palates immersed in a RingerR-diluted L-NAME solution at 120 ppm (n=10), 180 ppm (n=10) and 240 ppm (n=10); there was a control group (n=9) consisting of palates immersed in a RingerR solution only.

A similar procedure was applied to expose all palates: immersion in a RingerR solution or in one of the aminoguanidine or L-NAME solutions during four consecutive 15-minute periods. Mucociliary transport was assessed before and after each of the palate immersion periods, according to the proposed technique.^19^ Mucociliary transport was established by measuring the autologous mucus sample displacement velocity on the surface of mucus-depleted frog palates. Mucociliary transport velocity was established by measuring the time taken for mucus to move from the anterior to the posterior portion of the palate, using a stereoscope equipped with a measurement scale with a grid in one of its eyepieces.^3^ Samples of mucus were immersed in ethyl ether to remove the mineral oil before being placed on palate surfaces.^18^ Five measurements were made of each velocity to minimize errors.^20^ Palates remained inside the acrylic chamber at 100% humidity and temperature of 24°C during the measurements.

The final results were expressed as relative transport velocities, obtained by dividing the recorded palate transport velocity at 15, 30, 45 and 60 minutes by the recorded velocity at time zero before immersions (the baseline velocity).^19^

This study project was accepted by the Institutional Review Board on animal experimentation (number 41/05).

The mean relative transport velocities in all groups were compared based on the one-way analysis of variance (ANOVA) and by applying the Newman-Keuls multiple comparisons test for characterizing differences among results for each of the solutions and their concentrations, as well as the differences in immersion times for any same solution. The significance level was 5%.

## RESULTS

The transport velocity in palates immersed in RingerR's solution (controls) was statistically similar to baseline values across the transport velocity evaluation period, that is, after each immersion in RingerR's solution.

**Figure 1:**
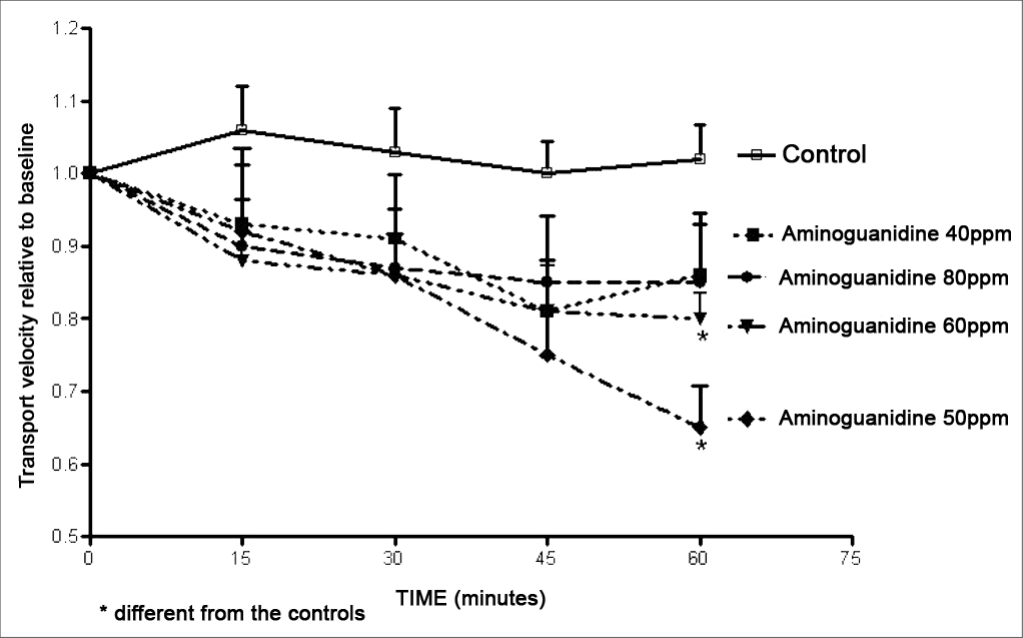
Relative velocity of mucociliary transport in frog palates immersed in Ringer^R^'s solution (controls) or in 40 ppm, 50 ppm, 60 ppm or 80 ppm aminoguanidine solutions.

Aminoguanidine decreased the mucociliary transport velocity, which appeared to be time-related, albeit not statistically significant. In the four aminoguanidine solutions of the experiment, the 50 ppm and the 60 ppm solutions showed decreased mucociliary transport velocity compared to controls (Fig. 1).

**Figure 2:**
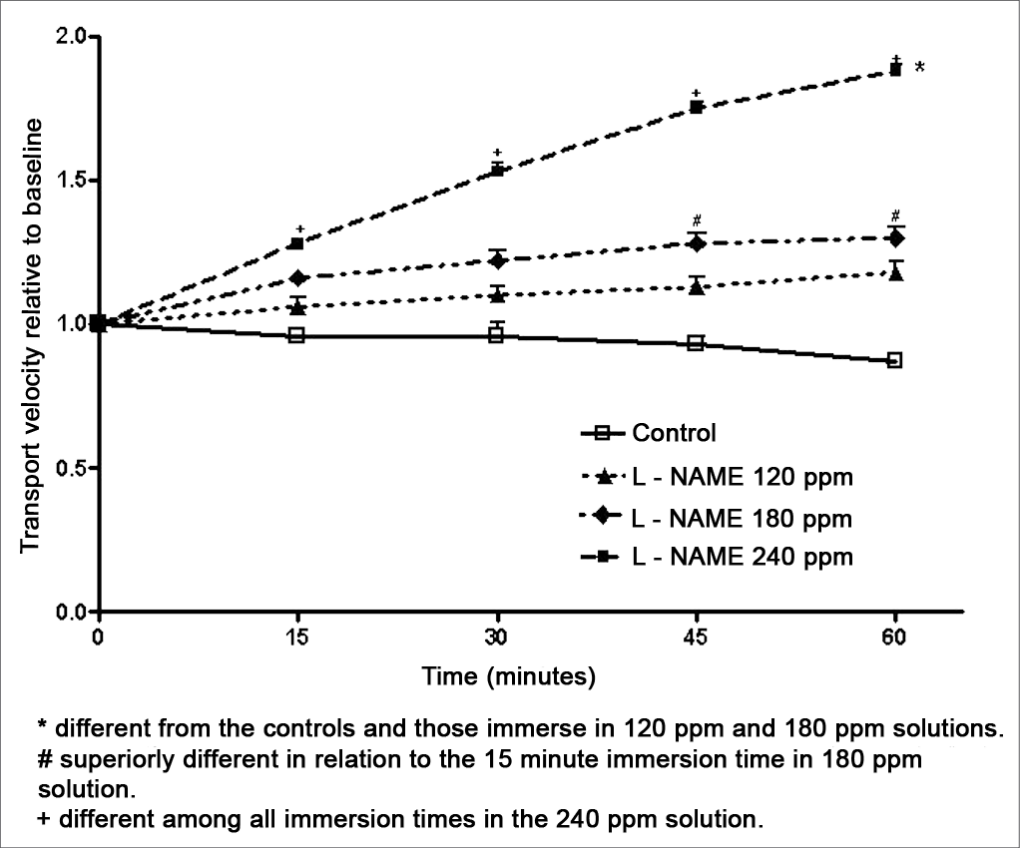
Relative velocity of mucociliary transport in frog palates immersed in RingerR's solution (controls) or in L-NAME solutions.

In opposition to aminoguanidine, L-NAME increased the mucociliary transport velocity. The L-NAME 120 and 180 ppm solutions did not increase the transport velocity significantly compared to controls. Velocity, however, increased with immersion time in an L-NAME 180 ppm solution, with a statistical difference between the 15-minute and the 45 and 60-minute immersion periods. A significant increased in transport velocity occurred in palates immersed in the L-NAME 240 ppm solution, compared to controls and to palates immersed in a 120 ppm solution. For immersion times, velocities recorded after 15, 30, 45 or 60 minutes were statistically different in palates immersed in a 240 ppm solution (Fig. 2).

## DISCUSSION

In this study we decided to study mucociliary transport in the frog palate; this palate is ciliated and secretes mucus, similar to human airways.1,3 This approach attempted to assess NO and NO-production pathway (cNOS and iNOS) involvement in mucociliary transportability. Our study showed that using L-NAME to inhibit the NO production constitutive pathway resulted in increased mucociliary transport velocity, and that aminoguanidine-induced inhibition of the inductive pathway resulted in decreased transport times.

Several studies have demonstrated NO involvement in ciliary activity. Studies of the rabbit maxillary sinus have shown that L-arg increases the ciliary beat frequency, and that this effect is decreased after NOS is blocked by N(G)-nitro L-arginine (L-NNA).21 Ciliary stimulation by NO-mediated transmitter or mediator substances has shown that NO operates as an intermediate messenger responding to these stimuli in the ciliated epithelium, but that NO-dependent mechanisms did not constitute a single pathway for stimulating ciliary function.22 Subsequently, sodium nitroprussiate (an NO donor) was found to decrease the saccharine transport time, while L-NAME was found to decrease nasal NO and to prolong the saccharine transport time in healthy subjects pre-treated with an anticholinergic drug.13

Results of a study done with L-arg and L-NAME have suggested that NO may have a regulating function on ciliary motility in the paranasal sinus mucosa of healthy subjects, and that eNOS and iNOS produce NO in healthy mucosae; it appeared further that eNOS seemed to have a more important role in producing NO.23

In our study we found that L-NAME increased mucociliary transport velocity in frog palates compared to velocities before immersing these palates in an L-NAME solution. This differs from the proposition that L-NAME decreases ciliary activity. It should be noted that L-NAME, being a non-specific cNOS inhibitor, and depending on the dose, may also block iNOS action.24,9 We used L-NAME and aminoguanidine to block cNOS and iNOS in turn, obtaining different and antagonic results, which suggests that blockage was selective, and that the mucociliary transport role of NO depends on which enzymatic pathway is activated.

Other studies of NO action have shown different results. Instillation of a lipopolysaccharide (E coli cell wall component that sets infection processes in motion) in the nasal cavity of guinea-pigs resulted in significantly increased NO production, which was harmful for the respiratory ciliated epithelium by causing damage to ciliated epithelial cells and decreasing the ciliary beat frequency.25 It has been suggested that in otitis media, the contradictory action of NO may be explained by different NO reactions in specific biological conditions. Low NO production in physiological conditions may have regulating or anti-inflammatory functions. In pathological conditions - such as inflammation - iNOS is activated and NO production increases.26 In the maxillary sinuses of healthy subjects, however, iNOS-expressed NO production was thought to support ciliary function and immune defenses, while in rhinosinusitis and septicemia, NO production decreased due to reduced iNOS activity, which led to compromised local defenses and an increased risk of secondary infections.^15^

It has subsequently been proposed that under normal conditions, NO is produced mainly by the iNOS pathway in nasal sinus epithelial cells, and under inflammatory conditions, NO is produced by the iNOS pathway in inflammatory cells. The iNOS activity in sinus epithelium appears to be essential for constant NO production, which is needed for maintaining ciliary beats at an optimal frequency for ideal mucociliary clearance, thus keeping the sinuses healthy. In rhinosinusitis, iNOS expression in epithelial cells decreases, but NO production by iNOS in host defense cells in the nasal cavity increases significantly. In such a process, significant amounts of NO and its metabolites may have a fundamental role in the pathogenesis of rhinosinusitis.^27^

Our study showed that the mucociliary transport velocity in palates immersed in an aminoguanidine solution decreased, compared to the mean velocity before immersion. Concentrations of 50 ppm and 60 ppm were the most effective inhibitory doses, suggesting dose adaptation for the enzyme inhibitory response in frog palates. Our results confirm that in healthy frog epithelium, iNOS-produced NO promotes mucociliary transport, since iNOS inhibition by aminoguanidine decreased transport velocity.

## CONCLUSION

Non-specific blockage of cNOS by L-NAME and a relatively specific blockage of iNOS by aminoguanidine allowed us to propose that, depending on the production pathway, NO may increase or decrease mucociliary transport in frog palates, suggesting a double role for NO in mucociliary transport in this epithelium.
